# Association between 15 insertion/deletion genetic polymorphisms and risk of schizophrenia using pooled samples

**DOI:** 10.17179/excli2022-5734

**Published:** 2023-02-28

**Authors:** Maedeh Bordbar, Mostafa Saadat

**Affiliations:** 1 Department of Biology, College of Sciences, Shiraz University, Shiraz 71467-13565, Iran

**Keywords:** insertion/deletion polymorphisms, schizophrenia, pooled samples

## Abstract

Schizophrenia is a psychiatric syndrome that affects approximately 1 % of the world population and is among the top 10 reasons for disability. In this case-control study, we investigated the association between 15 insertion/deletion (Indel) polymorphisms and schizophrenia risk using pooled samples. In the present case-control study, 361 individuals with schizophrenia and 360 healthy individuals were included in the study. We examined the insertion/deletion polymorphisms in *APOB*, *ADRA2B*, *PDCD6IP*, *LRPAP1*, *TLR2*, *DHFR*, *VEGF*, *HLA-G*, *TPA*, *DBH*, *UCP2*, *FADS2*, *MDM2*, *TP53* and *SLC6A4* genes. Our results revealed that the Del allele of the *HLA-G* 14bp Indel polymorphism increased the risk of schizophrenia (OR=1.23, 95 % CI=1.01-1.52, p=0.045) and the Alu^-^ allele of the *TPA *Alu^+^/Alu^-^ polymorphism negatively associated with the schizophrenia risk (OR=0.67, 95 % CI=0.54-0.82, p<0.001).

## Introduction

Schizophrenia is a common severe mental illness. Twin and other studies have consistently shown that there is a large genetic component to schizophrenia, with heritability estimated at around 80 %. Genome-wide association studies showed that multiple common variants, each with small effect, are associated with schizophrenia. More than 100 loci are significantly associated with schizophrenia (McCutcheon et al., 2020[4]). 

Both single nucleotide polymorphisms (SNPs) and Insertion/deletions (Indels) generally are abundant in human genomes. Although, Indels discovery efforts have lagged significantly behind SNPs discovery efforts, numerous Indels have been identified. Many of Indels have been identified within known genes and they might be located in the promoters and exons of genes, where gene function would be expected to be influenced the greatest (Mills et al., 2006[5]). Taken together, Indels are an important source of genetic variations. It should be noted that there are no so many reports on association between Indels and the risk of multifactorial complex diseases including schizophrenia. Therefore, the present study was carried out to examine the association between several Indels and schizophrenia risk using pooled samples. 

*APOB* 9bp Indel, *ADRA2B* 9bp Indel, *PDCD6IP* 15bp Indel, *LRPAP1* 37bp Indel, *TLR2* 22bp Indel, *DHFR *19bp Indel, *VEGF* 18bp Indel, *HLA-G* 14bp Indel, *TPA* 311bp Indel (also named Alu^+^/Alu^-^ polymorphism), *DBH* 19bp Indel, *UCP2* 45bp Indel, *FADS2* 22bp Indel, *MDM2* 40bp Indel, *TP53* 16bp Indel and *SLC6A4* 44bp Indel (also named L/S polymorphism) were studied. 

## Materials and Methods

### Participants

In the present case-control study, 361 individuals with schizophrenia (267 males, 94 females; mean age ± SD = 41.5 ± 13.0 years) and 360 healthy individuals (267 males, 93 females; mean age ± SD = 39.8 ± 11.2 years) were included in the study, as reported previously (Taghipour et al., 2019[11]; Abbasi and Saadat, 2021[1]). As mentioned in our previous reports, all participants were Caucasians living in Shiraz (Fars province, southern Iran). A psychiatrist performed Diagnostic and Statistical Manual of Mental Disorders, fourth edition (DSM-IV) Clinical Interview and the patients were chronic cases. The present case-control study has been approved by Shiraz University's ethics committee. Informed consent has been taken from all individuals under the study. 

Using the Epi Info software (version 7.2.2.6), assuming a statistical power of 0.80, α=0.05, case/control ratio equal to 1.0, 20 % frequency of the Del alleles for Indel polymorphisms, and OR=1.50, a minimum of 270 patients and 270 controls would be needed to identify a real difference in allelic frequency between the schizophrenia patient and healthy control groups. It should be noted that here 361 schizophrenia and 360 controls were included in the present case-control study.

### Estimation of allelic frequencies

Considering that the use of pooled samples for estimation of allelic frequencies is simple, inexpensive and fast method, this method was used in the present study to compare allelic frequencies of 15 Indels polymorphisms between the schizophrenia patients and control group. Genomic DNA was extracted from blood samples by boiling method (Newton, 1995[6]). Two pooled samples (healthy control and schizophrenia groups) were prepared by mixing equal amounts of extracted genomic DNA. As mentioned previously (Saadat et al., 2019[10]), the accuracy of relationship between the band intensity and DNA amount was investigated using serial dilution samples. Using 1, 2, 4, 8, and 16 dilution times of a single PCR product, the correlation coefficient between the band intensity and the relative DNA amount was investigated, the slope of linear regression (in no-intercept model) was equals to 0.998. This slope is showing the efficiency of the model for estimation of the relative DNA amount. It should be noted that the band intensity has been measured with ImageJ software, version 1.53e. 

The pooled DNA samples and DNA of a heterozygous individual (as a calibrator sample) were used for PCR. The specific forward and reverse primers for PCR were shown in the Supplementary information. After PCR, on gel electrophoresis, two bands were observed for the Ins and Del alleles. The intensity of the Ins and Del bands in pooled samples and heterozygote sample, was measured and the allelic frequencies in pooled samples were estimated using the method described previously (Saadat et al., 2019[10]). PCR, gel electrophoresis, and measuring the intensity of the bands were performed three times. For each polymorphism, the number of Ins and Del alleles in schizophrenia and control groups were calculated using the estimated allelic frequencies. 

### Statistical analysis

The ORs (odds ratios) and the 95 % confidence intervals (95 % CIs) indicate the association between polymorphisms and schizophrenia. In statistical comparisons, the Ins alleles were used as reference alleles (OR=1.0). Data was analyzed by SPSS software version 25. The logistic regression was used and the statistical significance level was set at a p-value of 0.05.

## Results and Discussion

Data on allelic frequencies of the studied Indel genetic polymorphisms in schizophrenia patients and control group were summarized in Table 1(Tab. 1). The results showed that the Del allele of the HLA-G 14bp Indel polymorphism increased the risk of schizophrenia (OR=1.23, 95 % CI=1.01-1.52, p=0.045) and the Alu^-^ allele of the TPA Alu+/Alu- polymorphism negatively associated with the schizophrenia risk (OR=0.67, 95 % CI=0.54-0.82, p<0.001). 

However, it should be noted that significant differences were not observed in the other insertion/deletion polymorphisms studied in this research (Table 1(Tab. 1)). 

In this study, we used a simple, fast, inexpensive, and accurate method for estimating the allelic frequency of 15 insertion/deletion polymorphisms in pooled samples so that we could examine more genes in the appropriate sample size in a short time. 

The TPA Alu^+^/Alu^-^ is a functional polymorphism and might play a role in the protein secretion rate or protein primary structure (Jern et al., 1999[3]). However, there is no study on this polymorphism and the risk of schizophrenia, but previous studies showed that abnormal function of TPA is related to the pathogenesis of schizophrenia and tPA actively participates in the mechanisms of neurogenesis and angiogenesis, which might justify not only the impaired neurogenesis but also the low prevalence of neoplastic diseases among schizophrenics (Hoirisch-Clapauch and Nardi, 2013[2]). The present study shows that the insertion allele is associated with an increased risk of schizophrenia.

In addition, the human chromosome segment 6p21.1-p22.3 has a significantly higher number of susceptible loci, including the HLA, suggesting that components of the immune system are associated with the risk of schizophrenia (Saadat, 2013[9]). *HLA-G* 14bp Indel is a functional polymorphism and is associated with the gene expression. This genetic variant also influences brain morphometric measures and HLA-G could be an important biomarker for schizophrenia (Rajasekaran et al, 2014[8]). Further, low levels of soluble HLA-G were shown to have a significant impact on severity of schizophrenia (Rajasekaran et al, 2016[7]). Our study demonstrates that the deletion allele is associated with an increased risk of schizophrenia.

Finally, we should mention major limitations of the present study. By comparing the allelic frequency between schizophrenia and control groups using pooled samples, we cannot compare genotypes of the cases and controls with each other and investigate the effects of environmental factors and genotypes simultaneously. Also, it is impossible to study the potential interactive effects of associate genes. As mentioned earlier, the use of pooled sample method is accompanied by a reduction in costs and time, and the researcher can simply examine a larger number of genetic polymorphisms.

In summary, the HLA-G 14bp Indel and tPA Alu Indel polymorphisms were associated with schizophrenia. Future case-control genetic association studies are needed to conclude that these polymorphisms are risk factors for schizophrenia. 

## Declaration

### Ethical statement 

Ethical approval was obtained from Shiraz University Ethics Research Committee.

### Disclosure of potential conflicts of interest

The authors declare no conflicts of interest.

### Acknowledgments

Authors are indebted to the participants for their close cooperation. 

## Supplementary Material

Supplementary information

## Figures and Tables

**Table 1:**
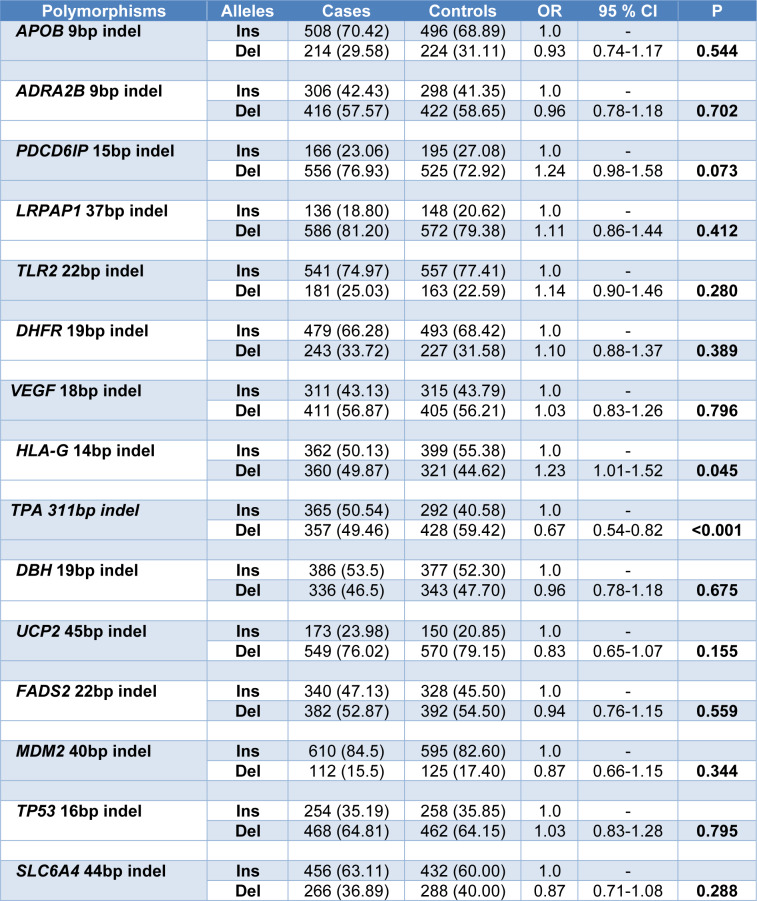
Comparison of allelic frequency of 15 insertion/deletion genetic polymorphisms in control and schizophrenia groups
