# Supplementary material for: Association between 15 insertion/deletion genetic polymorphisms and risk of schizophrenia using pooled samples
Source: EXCLI J. 2023 Feb 28;22:310–4. doi: 10.17179/excli2022-5734 (PMC10201014; doi:10.17179/excli2022-5734)
Supplement: Supplementary information [file EXCLI-22-310-s-001.pdf]

EXCLI Journal 2023;22:310-314 – ISSN 1611-2156
Received: December 24, 2022, accepted: February 21, 2023, published: February 28, 2023

Supplementary information to:

Original article:

ASSOCIATION BETWEEN 15 INSERTION/DELETION GENETIC POLYMORPHISMS AND RISK OF SCHIZOPHRENIA USING POOLED SAMPLES

Maedeh Bordbar, Mostafa Saadat*

Department of Biology, College of Sciences, Shiraz University, Shiraz 71467-13565, Iran

* **Corresponding author:** Mostafa Saadat, Department of Biology, College of Sciences, Shiraz University, Shiraz 71467-13565, Iran. Fax: +98-71-32280926.
E-mail: saadat@shirazu.ac.ir

https://dx.doi.org/10.17179/excli2022-5734

This is an Open Access article distributed under the terms of the Creative Commons Attribution License (http://creativecommons.org/licenses/by/4.0/).

EXCLI Journal 2023;22:310-314 – ISSN 1611-2156
Received: December 24, 2022, accepted: February 21, 2023, published: February 28, 2023

Supplementary Table 1: The specific forward and reverse primers and PCR product length of each indel polymorphism

Loci	Primer sequences	PCR product length (bp)	Reference
APOB	**F:** 5′ ACCGGCCCTGGCGCCCGCCAGCA 3′<br>**R:** 5′ CAGCTGGCGATGGACCCGCCGA 3′	93/84	Boerwinkle and Chan, 1989
ADRA2B	**F:** 5′ CAAGCTGAGGCCGGAGACACT 3′<br>**R:** 5′ AGGGTGTTTGTGGGGCATCT 3′	112/103	Eldeeb et al, 2021
PDCD6I	**F:** 5′ CCTTAAGGTCTGTGTCAACC 3′<br>**R:** 5′ TATTCCTCCACTCGAACAAC 3′	227/212	Liu et al., 2014
LRPAP1	**F:** 5′ AGTGTGCGTGGAGCCTATG 3′<br>**R:** 5′ GGTGTTTCTGGACACAAAGGA 3′	222/185	Benes et al., 2000
TLR2	**F:** 5′ CACGGAGGCAGCGAGAAA 3′<br>**R:** 5′ CTGGGCCGTGCAAAGAAG 3′	286/264	Tahara et al., 2007
DHF	**F:** 5′ CGCAAGTCTGGCCCCATC 3′<br>**R:** 5′ TCAGGTATCTGCCGGGCC 3′	119/100	Hayashi et al., 2010
VEGF	**F:** 5′ GCTGAGGATGGGGCTGACTAGGTA 3′<br>**R:** 5′ GTTTCTGACCTGGCTATTTCCAGG 3′	230/212	Buraczynska et al.,2007
HLA-G	**F:** 5′ GTGATGGGCTGTTTAAAGTGTCACC 3′<br>**R:** 5′ GGAAGGAATGCAGTTCAGCATGA 3′	224/210	Hviid et al., 1999
TPA	**F:** 5′ GTAAGAGTTCCGTAACAGGACAGCT 3′<br>**R:** 5′ CCCCACCCTAGGAGAACTTCTCTTT 3′	424/113	Valle-Garay et al., 2013
DBH	**F:** 5′ TGCAAAAATCAGGCACATGC 3′<br>**R:** 5′ TCCAATAATTTGGCCTCAATC 3′	166/147	Barbanti et al., 2019
UCP2	**F:** 5′ CAGTGAGGGAAGTGGGAGG 3′<br>**R:** 5′ GGGGCAGGACGAAGATTC 3′	502/457	Wang et al., 2007
FADS2	**F:** 5′ TTTCTCAAAGGCCGTGGTGT 3′<br>**R:** 5′ AGTGCTAACCACTCCTGGAA 3′	629/607	Kothapalli et al., 2016
MDM2	**F:** 5′ TTTCCTTTCTGGTAGGCTGG 3′<br>**R:** 5′ CACCTACTTTCCCACAGAGA 3′	262/222	Lalonde et al., 2012
TP53	**F:** 5′ TCAAATCATCCATTGCTTGG 3′<br>**R:** 5′ TGGGACTGACTTTCTGCTCTT 3′	195/179	Wu et al., 2002
SLC6A4	**F:** 5′ GACATAATCTGTCTTCTGGCCTCTCAAG 3′<br>**R:** 5′ CAATGTCTGGCGCTTCCCCTACATAT 3′	310/266	Hauser et al., 2003

Received: December 24, 2022, accepted: February 21, 2023, published: February 28, 2023

REFERENCES

Barbanti P, Guadagni F, De Marchis ML, Ialongo C, Egeo G, Fofi L, et al. Dopamine-beta-hydroxylase 19-bp insertion/deletion polymorphism affects medication overuse in patients with chronic migraine. Neurol Sci. 2019;40:1717-24.

Benes P, Muzik J, Benedík J, Elbl L, Znojil V, Vácha J. Relation between the insertion/deletion polymorphism in the gene coding for receptor associated protein (RAP) and plasma apolipoprotein AI (apoAI) and high-density lipoprotein cholesterol (HDL) levels. Clin Genet. 2000;57:309-10.

Boerwinkle E, Chan L. A three codon insertion/deletion polymorphism in the signal peptide region of the human apolipoprotein B (*APOB*) gene directly typed by the polymerase chain reaction. Nucleic Acids Res. 1989;17:4003.

Buraczynska M, Ksiazek P, Baranowicz-Gaszczyk I, Jozwiak L. Association of the *VEGF* gene polymorphism with diabetic retinopathy in type 2 diabetes patients. Nephrol Dial Transplant. 2007;22:827-32.

Eldeeb HM, Elgharabawy RM, Abd Elmoniem AE, Ahmed AA. Alpha-2 beta-adrenergic receptor (301–303 I/D) gene polymorphism in hypertension and type 2 diabetes mellitus diseases among Saudi cases in the Qassim region. Sci Prog. 2021;104: 368504211012162.

Hauser J, Leszczyńska A, Samochowiec J, Czerski PM, Ostapowicz A, Chlopocka M, et al. Association analysis of the insertion/deletion polymorphism in serotonin transporter gene in patients with affective disorder. Eur Psychiatry. 2003;18:129-32.

Hayashi H, Horino M, Morishita M, Tazoe Y, Tsuboi S, Matsuyama T, et al. Dihydrofolate reductase gene intronic 19-bp deletion polymorphisms in a Japanese population. Drug Metab Pharmacokinet. 2010;25:516-8.

Hviid TV, Sørensen S, Morling N. Polymorphism in the regulatory region located more than 1.1 kilobases 5′ to the start site of transcription, the promoter region, and exon 1 of the *HLA-G* gene. Hum Immunol. 1999; 60:1237-44.

Kothapalli KS, Ye K, Gadgil MS, Carlson SE, O’Brien KO, Zhang JY, et al. Positive selection on a regulatory insertion–deletion polymorphism in *FADS2* influences apparent endogenous synthesis of arachidonic acid. Mol Biol Evol. 2016;33:1726-39.

Lalonde M-E, Ouimet M, Larivière M, Kritikou EA, Sinnett D. Identification of functional DNA variants in the constitutive promoter region of *MDM2*. Hum Genomics. 2012;6:15.

Liu SG, Yuan SH, Wu HY, Huang CS, Liu J. The programmed cell death 6 interacting protein insertion/deletion polymorphism is associated with non-small cell lung cancer risk in a Chinese Han population. Tumor Biol. 2014;35:8679-83.

Tahara T, Arisawa T, Wang F, Shibata T, Nakamura M, Sakata M, et al. Toll-like receptor 2 –196 to 174 del polymorphism influences the susceptibility of Japanese people to gastric cancer. Cancer Sci. 2007;98: 1790-4.

Valle-Garay E, Montes AH, Corte JR, Meana A, Fierer J, Asensi V. tPA Alu (I/D) polymorphism associates with bacterial osteomyelitis. J Infect Dis. 2013; 208:218-23.

Wang X, Axelsson J, Nordfors L, Qureshi AR, Avesani C, Barany P, et al. Changes in fat mass after initiation of maintenance dialysis is influenced by the uncoupling protein 2 exon 8 insertion/deletion polymorphism. Nephrol Dial Transplant. 2007;22:196-202.

Wu X, Zhao H, Amos CI, Shete S, Makan N, Hong WK, et al. p53 genotypes and haplotypes associated with lung cancer susceptibility and ethnicity. J Natl Cancer Inst. 2002;94:681-90.
